# Development of a five-protein signature for predicting the prognosis of head and neck squamous cell carcinoma

**DOI:** 10.18632/aging.104036

**Published:** 2020-10-13

**Authors:** Xinyuan Zhao, Xianwen Liu, Li Cui

**Affiliations:** 1 Stomatological Hospital, Southern Medical University, Guangzhou 510280, China; 2 UCLA School of Dentistry, Los Angeles, CA 90095, USA

**Keywords:** head and neck squamous cell carcinoma, prognostic signature, survival analysis, HER3_pY1289, the cancer proteome atlas

## Abstract

Currently no reliable indicators are available for predicting the clinical outcome of head and neck squamous cell carcinoma (HNSCC). This study aimed to develop a protein-based model to improve the prognosis prediction of HNSCC. The proteome data of HNSCC cohort was downloaded from The Cancer Proteome Atlas (TCPA) portal. The TCPA HNSCC cohort was randomly divided into the discovery and validation cohort. A protein-based risk signature was developed with the discovery cohort, and then verified with the validation cohort. The prognostic value of HER3_pY1289 was further determined. We have constructed a five-protein risk signature which was strongly associated with the overall survival (OS) in the discovery cohort. Similar findings were observed in the validation cohort. The protein-based risk signature was identified as an independent prognostic factor for HNSCC. A nomogram model built on the protein-based risk signature exhibited good performance for predicting OS. Our immunohistochemistry (IHC) analysis showed that higher HER3_pY1289 staining intensity was closely associated with unfavorable prognosis of HNSCC. HER3 suppression inhibited the proliferation and invasion capacity of HNSCC cells. Collectively, we have developed a protein-based risk signature for accurately predicting the prognosis of HNSCC, which might provide valuable information for optimal individualized treatment regimens.

## INTRODUCTION

Head and neck cancer (HNC) represents the sixth most common cancer worldwide, and squamous cell carcinoma (SCC) consist of more than 90% of HNC [1]. Accumulation of genetic alterations, exposure to environmental risk factors, viral infection and unhealthy lifestyles contribute to the initiation and progression of head and neck squamous cell carcinoma (HNSCC) [2]. Although great progress has been achieved for the therapeutic methodologies, the clinical outcome of HNSCC remains unsatisfactory in the past few decades [3]. Stratifying the HNSCC patients with different prognosis is of great importance for selecting the optimal therapeutic strategies for individual patient. Unfortunately, currently no reliable indicator is available for accurately predicting the prognosis of HNSCC. Therefore, development of robust models for distinguishing the HNSCC patients with different risks is urgently needed.

The recent development of next-generation sequencing technology has significantly enriched and expanded our understanding on the association between DNA (genomic level) or RNA (transcript level) and cancer progression. Many DNA or RNA based prognostic signatures have also been developed for predicting the prognosis of human cancer including HNSCC [4–6]. However, proteins are the basic functional units for executing the biological processes. In addition, most cancer therapy targeted the proteins instead of DNA and RNA. Moreover, poor correlation might be observed between DNA and RNA levels with protein levels [7]. Therefore, it is important for measuring the protein levels directly and explore their associations with the clinical outcome of HNSCC. Reverse-phase protein arrays (RPPAs) is a powerful proteomic approach for assessing the levels of interested proteins across different samples in a high-throughput manner [8, 9]. The Cancer Proteome Atlas (TCPA) is a public accessible platform which spans more than 8,000 patient samples through The Cancer Genome Atlas (TCGA). In addition, approximately 300 protein markers have been examined in the tumor samples [10, 11]. Therefore, TCPA is highly valuable resource for developing protein-based risk signature for predicting the clinical outcome of HNSCC.

In this study, the TCPA HNSCC cohort was randomly divided into discovery cohort and validation cohort. A five-protein risk signature was developed with the discovery cohort, and then successfully verified in the validation cohort. In addition, a nomogram model based on the protein-based risk signature was constructed and it demonstrated impressive predictive accuracy for overall survival (OS) of HNSCC. Our immunohistochemistry (IHC) analysis showed that the staining intensity of HER3_pY1289, a protein in the risk signature, was positively associated with unfavorable clinical outcome of HNSCC. The expression level of HER3_pY1289 was markedly reduced following downregulation of HER3, and knockdown of HER3 inhibited the oncogenic activities of HNSCC cells.

## RESULTS

### Candidate OS-related proteins of HNSCC patients in the TCPA discovery cohort

**Figure 1:**
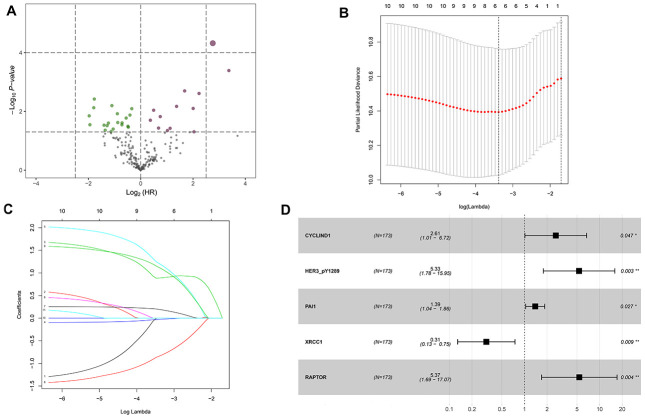
**Identification of the overall survival (OS)-associated proteins in the TCPA discovery cohort.** (**A**) Volcano plot of proteins that are significantly associated with OS of HNSCC. Y-axis indicates the *p* values (-log_10_ scale), whereas the X-axis shows the hazard ratio (log_2_ scale). Each symbol represents a different protein, and the dark red and green symbols categorize the risky (n=13) and protective (n=20) proteins, respectively. (**B**) Tuning parameter (logλ) selection cross-validation error curve for OS-associated proteins. The vertical dotted line is drawn at the optimal value by the minimum criteria and the 1-SE criteria. (**C**) The LASSO coefficient profile of 6 OS-related proteins and the vertical dotted line is drawn at the value chosen by 10-fold cross-validation. (**D**) Multivariate Cox proportional hazards regression analysis reveals five independent prognostic proteins of HNSCC patients in the discovery cohort.

A total of 33 proteins that significantly associated with OS are identified by the univariate cox proportional hazards regression analysis. The proteins with the hazard ratio (HR) larger than 1 are risky proteins, while those with the HR less than 1 are protective proteins. As shown in Figure 1A and Supplementary Table 1, 13 risky (dark red) and 20 protective (green) proteins are identified. Then the above 33 OS-associated proteins are subjected to least absolute shrinkage and selection operator (LASSO) analysis, which is a regression analysis methodology that performs both variable selection and regularization in order to enhance the prediction accuracy and interpretability of the statistical model. It is widely used for optimal selection of features in high-dimensional data with a strong predictive value and low correlation between one another to prevent overfitting. Therefore, it is highly efficient for identifying the most useful predictive markers and generating a prognostic signature for predicting clinical outcome. The dashed vertical line indicates the optimal value of log λ with the minimum partial likelihood deviance, and therefore 6 proteins (CyclinD1, E-cadherin, HER3_pY1289, PAI-1, XRCC1 and Raptor) are selected for the subsequent multivariate analysis (Figure 1B). The LASSO coefficient of the 6 proteins is shown in Figure 1C. Then multivariate cox proportional hazards regression analysis is performed to identify the independent prognostic proteins. The results show that CyclinD1, HER3_pY1289, PAI-1, XRCC1 and Raptor are the prognostic proteins independently associated with OS in the discovery cohort (Figure 1D). Subsequently, the independent prognostic proteins are used to build up a risk score model. A risk score for each patient is calculated as the sum of each protein’s score, which is obtained by multiplying the expression level of the protein and its corresponding coefficient. The coefficients of the proteins are obtained from the multivariate analysis. The following formula is developed to calculate risk score for each patient: risk score = (0.960×CyclinD1) + (1.673×HER3_pY1289) + (0.330×PAI-1) + (-1.169×XRCC1) + (1.681×Raptor).

### Construction of a protein-based prognostic model using the TCPA discovery cohort

**Figure 2:**
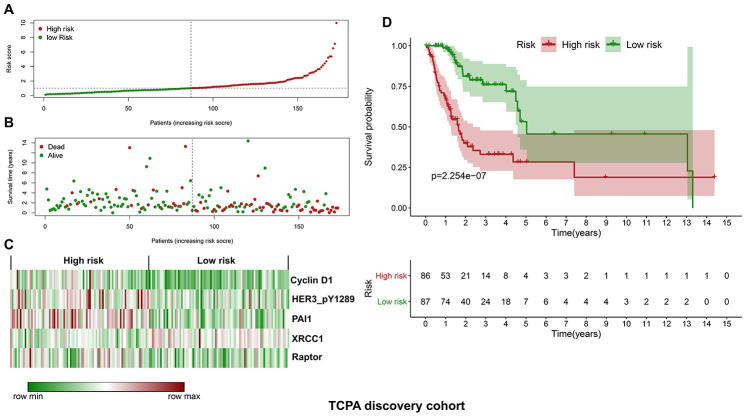
**Construction of a protein-based prognostic signature based on the TCPA discovery cohort.** (**A**) The distribution of risk scores in the low and high-risk groups. The risk scores for all patients in discovery cohort are plotted in ascending order and are divided by the threshold (vertical dotted line). The dots in the left (green) and right (dark red) side of the vertical dotted line belong to the low and high-risk groups, respectively. The risk scores are gradually increased from the low-risk group to high-risk group. (**B**) The pattern of survival time and survival status in low and high-risk groups. The dots in the left and right side of the vertical dotted line belong to the low and high-risk group, respectively. The dark red and green dots indicate death and survival, respectively. The high-risk group has a significantly higher mortality rate than the low-risk group. (**C**) The expression levels of the five prognostic proteins for each patient in the discovery cohort, with dark red indicating higher expression and green representing lower expression. (**D**) Survival analysis demonstrates that the patients in the high-risk group have statistically significant worse OS than those in low-risk group.

**Figure 3:**
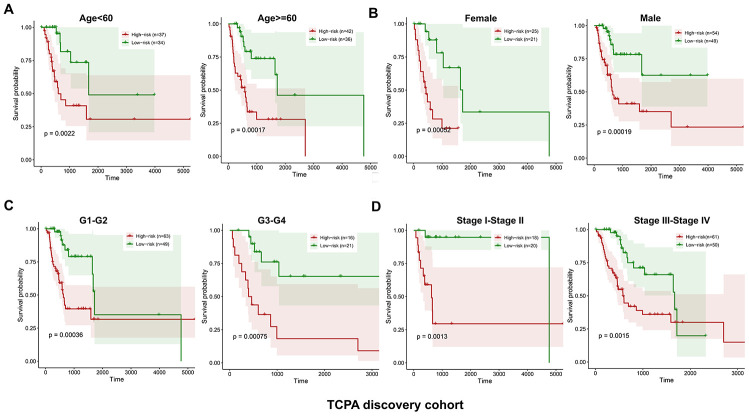
**Kaplan-Meier curves of OS differences stratified by age, gender, tumor grade or TNM stage between low and high-risk group in the TCPA discovery cohort.** The patients with low-risk scores have significantly better OS than patients with high-risk scores in different subgroups stratified by age (**A**), gender (**B**), tumor grade (**C**) and TNM stage (**D**).

The discovery cohort is divided into high risk group (n=86) and low risk group (n=87) using the median value of the risk scores. The distribution of risk scores in the high and low-risk groups is depicted in Figure 2A. Figure 2B shows the patterns of survival time and survival status between high and low-risk groups. The relative expression levels of the five prognostic proteins for each patient are shown in Figure 2C. The survival analysis demonstrates that the patients in the high-risk group have a significant shorter OS than the patients in the low-risk group (*p*=2.254e-07) (Figure 2D). The differences in OS stratified by the common clinicopathological parameters are further analyzed between high and low-risk groups. One hundred and forty-nine patients remain in the discovery cohort after removing the cases with missing information in age, gender, tumor grade or TNM stage. The clinical information of the discovery cohort is summarized in Supplementary Table 2. As shown in Figure 3A–3D, high-risk group has consistently worse OS than low-risk group for the subgroups stratified by age, gender, tumor grade or TNM stage.

### Validation of the protein-based prognostic model using the TCPA validation cohort

**Figure 4:**
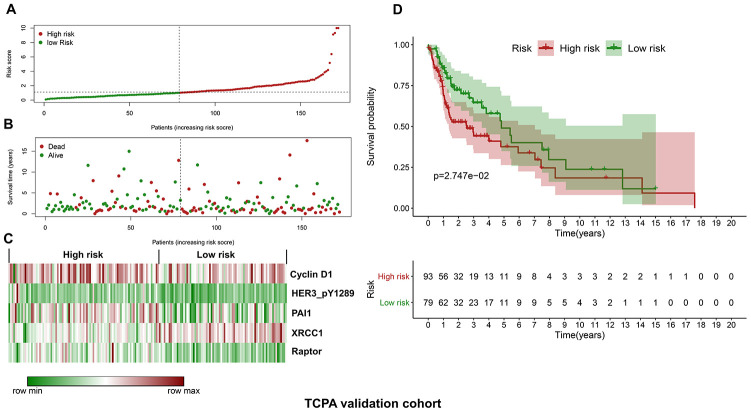
**Validation of the protein-based prognostic signature with the TCPA validation cohort.** (**A**) The distribution of risk scores in the low and high-risk groups. The risk scores for all patients in the validation cohort are plotted in ascending order and are divided by the threshold (vertical dotted line). The green and dark red dots belong to the low and high-risk groups, respectively. The risk scores are gradually increased from the low-risk group to high-risk group. (**B**) The pattern of survival time and survival status in low and high-risk groups. The dots in the left and right side of the vertical dotted line indicate the patients in the low and high-risk group, respectively. The dark red and green dots represent death and survival, respectively. The mortality rate is markedly higher in the high-risk group than in the low-risk group. (**C**) The expression levels of the five prognostic proteins for each patient in the validation cohort. The dark red color is indicative of higher relative expression and the green color represents lower expression (**D**) Survival analysis of the association between risk score and OS of HNSCC in the validation cohort. The OS is significantly shorter in patients in the high-risk group than those in the low-risk group.

**Figure 5:**
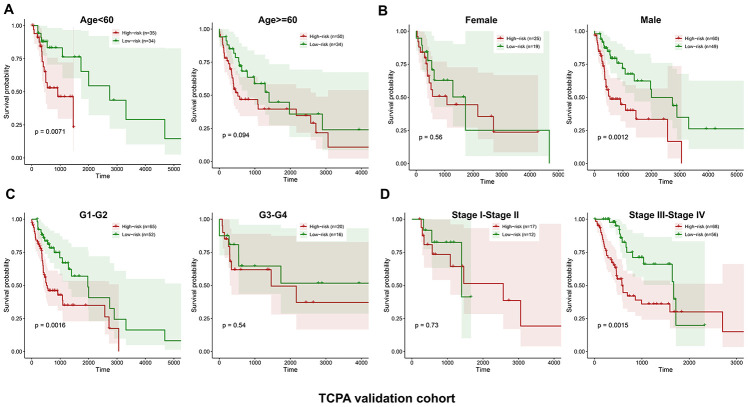
**Stratified analysis of the five-proteins signature for HNSCC patients in the TCPA validation cohort with age, gender, tumor grade or TNM stage.** The patients with high-risk scores have significantly worse OS than those with low-risk scores in the subgroups of age<60, male, grade 1-2 and stage III-IV. No significant difference in OS is found for the patients in the subgroups of age>=60, female, grade 3-4 and stage I-II (**A**–**D**).

Similarly, the validation cohort is divided into high-risk group (n=93) and low-risk group (n=79) using the median value of the risk scores generated in the discovery cohort. The distribution of risk scores as well as the distribution of survival time and survival status between high and low-risk groups are shown in Figure 4A and Figure 4B, respectively. The expression patterns of the five prognostic proteins in the validation cohort are revealed in Figure 4C. The high-risk group patients have poorer OS than the low-risk group patients (*p*=2.747e-02) (Figure 4D). One hundred and fifty-three patients remain in the validation cohort after removing the cases with missing information in age, gender, tumor grade or TNM stage. The clinical information of the validation cohort is described in Supplementary Table 3. For the cases with age<60, or male cases, or patients with G1-G2 or those at stage III-IV, the high-risk group patients suffer worse OS compared to the low-risk group. No significant difference in OS is found between high and low-risk groups for the patients with age>=60, or female cases, or patients with G3-G4, or those at stage I-II (Figure 5A–5D).

### The protein-based risk signature is an independent prognostic factor for HNSCC

**Figure 6:**
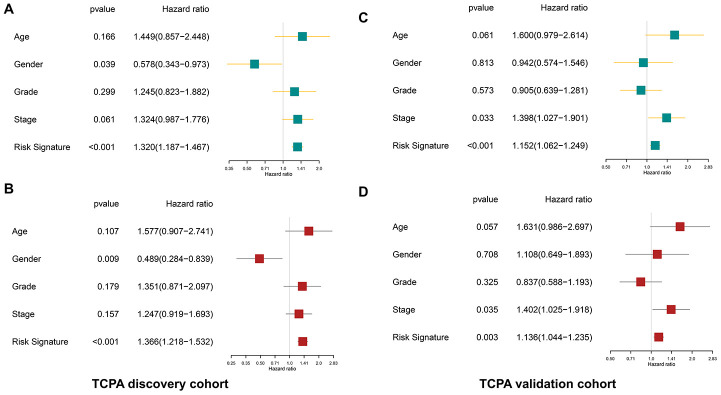
**The protein-based risk signature is an independent prognostic factor both in the discovery and validation cohort.** (**A**) The univariate analysis shows that the risk signature is significantly associated with OS of HNSCC in the discovery cohort. (**B**) The multivariate analysis demonstrates the risk signature is an independent prognostic factor in the discovery cohort. (**C**, **D**) Similar findings are observed in the validation cohort.

The clinicopathological parameters including age, gender, tumor grade, and TNM stage as well as the protein-based risk signature are subjected to the univariate and multivariate analyses. As shown in Figure 6A–6D, the univariate analysis reveals that the risk signature is significantly associated with the OS of HNSCC both in the discovery cohort (*p*<0.001, HR=1.320, 95% CI=1.187-1.467) and validation cohort (*p*<0.001, HR=1.152, 95% CI=1.062-1.249). The multivariate analysis shows that the risk signature is an independent prognostic factor both in the discovery cohort (*p*<0.001, HR=1.366, 95% CI=1.218-1.532) and validation cohort (*p*=0.003, HR=1.136, 95% CI=1.044-1.235).

### Nomogram model construction and prediction

**Figure 7:**
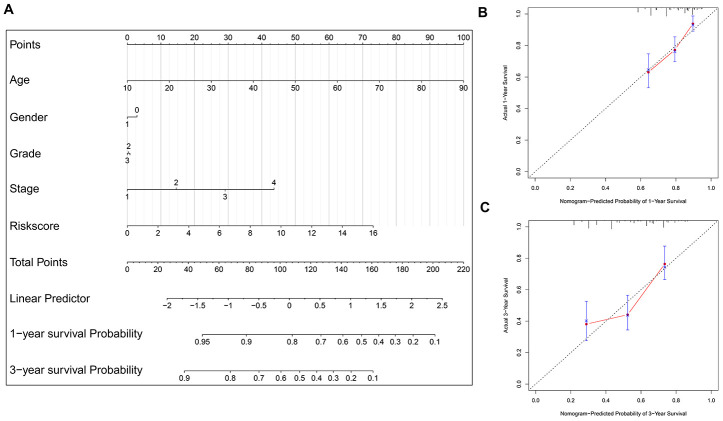
**Nomogram model construction and prediction.** (**A**) The nomogram model which includes the protein-based risk signature, age, gender, tumor grade and TNM stage is developed. The total nomogram score for each patient can be easily calculated based on the risk score and the clinicopathological parameters, which is then used to predict the 1-year and 3-year survival probability. (**B**, **C**) The calibration plots demonstrate that the nomogram model exhibits good predictability for 1-year OS and 3-year OS.

To facilitate the potential clinical application, a more sensitive nomogram predictive model is developed. As shown in Figure 7A, the risk signature, age, gender, tumor grade and TNM stage are included into the nomogram model to predict the prognosis of HNSCC. A nomogram-based score is calculated for each patient based on their risk scores and the clinicopathological parameters on the point scale. The calibration curves show that the nomogram model exhibits excellent performance for predicting the 1-year OS and 3-year OS of HNSCC (Figure 7B–7C).

### HER3_pY1289 is overexpressed in HNSCC and its potential prognostic value

**Figure 8:**
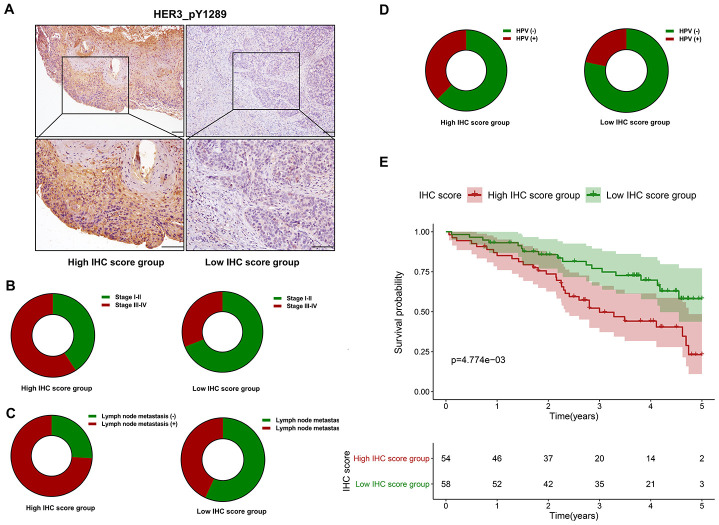
**The prognostic value of HER3_pY1289 in HNSCC.** (**A**) The staining intensity of HER3_pY1289 is higher in the high IHC score group compared to the low IHC score group (upper panels: scale bar=100 μm, lower panels: scale bar=100 μm). (**B**) A higher percentage of patients at the advanced stage is observed in the high IHC score group than in the low IHC score group. (**C**) A higher percentage of patients with positive lymph node metastasis is found in the high IHC score group. (**D**) A higher percentage of patients with positive HPV infection is found in the high IHC score group. (**E**) The survival analysis shows that the patients in the high IHC score group have worse OS than those in the low IHC score group.

Immunohistochemistry (IHC) is performed to evaluate the expression level of HER3_pY1289 in HNSCC samples. The IHC score is calculated for each sample in the SMUSH cohort. The median value of IHC score is used as the cut-off value to divide the SMUSH cohort into high IHC score group (n=54) and low IHC score group (n=58). Figure 8A shows the representative samples in the high and low IHC score group, and the staining intensity is significantly higher in high IHC score group compared to the low IHC score group. A higher percentage of HNSCC patients at the advanced stages (32/54 *vs* 18/58) or with positive lymph node metastasis (40/54 vs 25/58) or with human papillomavirus (HPV) infection (18/48 *vs* 10/46, 94 out of 112 HNSCC patients have the results of the HPV test) is found in the high IHC score group compared to the low IHC score group (Figure 8B–8D). The survival analysis reveals that the patients in the high IHC score group have markedly shorter OS than those in the low IHC score group (*p*=4.774e-03) (Figure 8E).

### Knockdown of HER3 suppresses the proliferation and invasion of HNSCC cells

**Figure 9:**
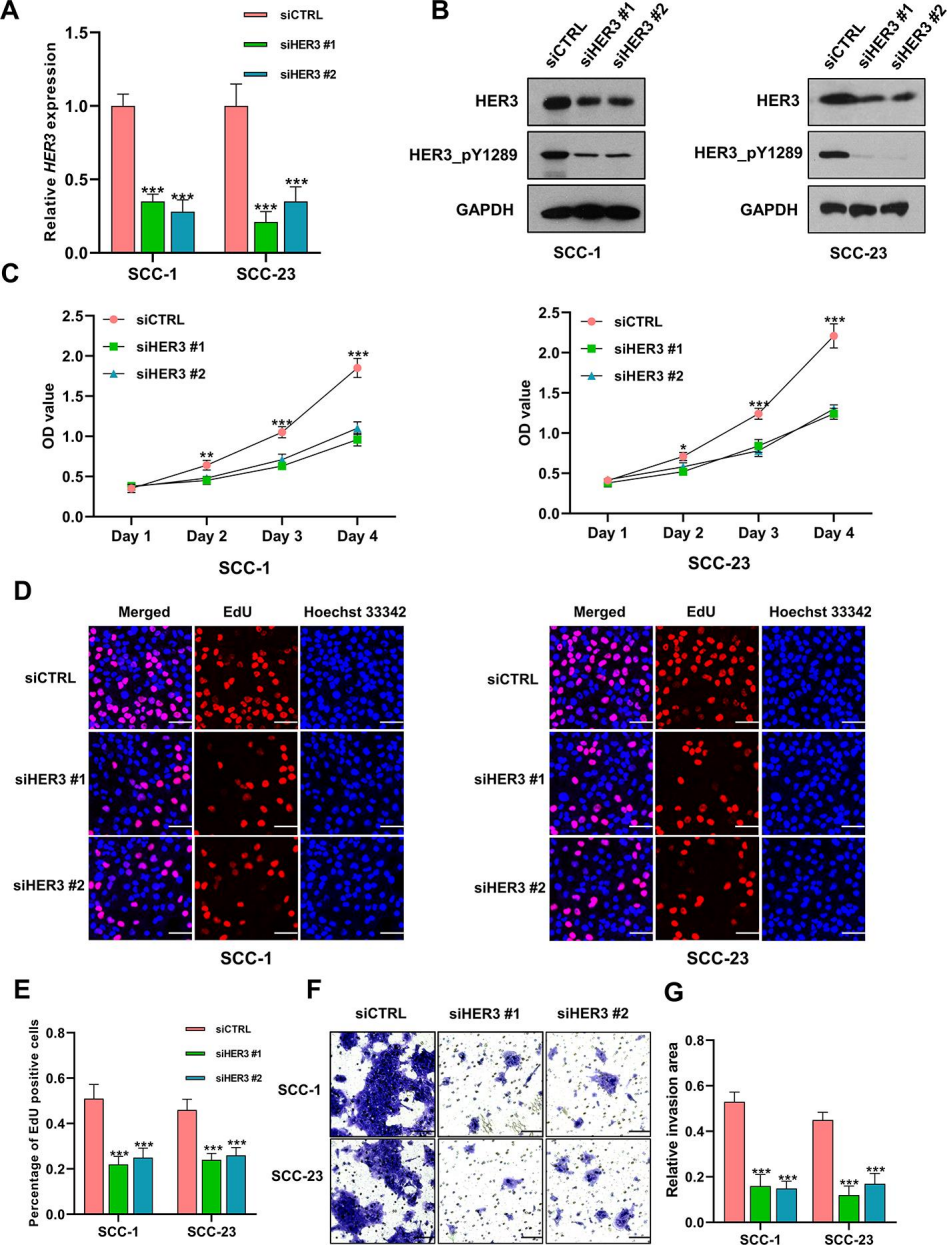
**Knockdown of HER3 suppresses the proliferation and invasion of HNSCC cells.** (**A**) The expression level of HER3 mRNA is significantly lower in siHER3 treated cells compared to the siCTRL treated cells. (**B**) Western blot shows that the levels of HER3 and HER3_pY1289 are lower in siHER3 treated cells compared to the siCTRL treated cells. (**C**) MTT assay reveals that the OD values are lower in the cells subjected to HER3 downregulation. (**D**, **E**) The EdU assay shows that the percentage of EdU positive cells is lower in siHER3 treated cells compared to the siCTRL treated cells (scale bar=20 μm). (**F**, **G**) The invasion assay shows the number of cells invading the membrane is lower in siHER3 treated group than in the control group (scale bar=100 μm).

The HER3 mRNA is markedly reduced in HNSCC cells following siHER3 transfections (Figure 9A). The western blotting results show that the expression levels of HER3 and HER3_pY1289 are significantly lower in siHER3 treated cells compared to the siCTRL treated cells (Figure 9B). The MTT assay reveals that the OD values are lower in HER3 knockdown cells at various time points (48h, 72h and 96h) compared to the control cells (Figure 9C). Similarly, the percentage of EdU positive cells is lower in HNSCC cells with HER3 downregulation (Figure 9D–9E). The number of HER3 knockdown cells that invaded through the membrane is significantly less than that of the control cells (Figure 9F, 9G).

## DISCUSSION

To the best of our knowledge, this is the first study to construct a protein-based prognostic signature for HNSCC with the TCPA dataset. A five protein-based risk signature was developed with the discovery cohort, and further validated in the validation cohort, indicating the risk signature was strongly associated with the OS of HNSCC. In addition, the multivariate cox model which included the common clinicopathological variables showed that the protein-based risk signature was an independent risk indicator for HNSCC both in the discovery and validation cohort. Moreover, the risk signature-based nomogram model exhibited good performance for predicting the OS of HNSCC, indicating it might have promising potential for clinical application. Our IHC analysis showed that high HER3_pY1289 staining intensity was positively correlated with aggressive clinical variables and unfavorable OS. Downregulation of HER3 suppressed the expression of HER3_pY1289 as well as the oncogenic activities of HNSCC cells. These results suggested that HER3_pY1289 played a tumor promoting role in HNSCC carcinogenesis, which further supported the findings that HER3_pY1289 was a risky protein in the risk signature model.

Cyclin D1 plays a critical role in regulating cell cycle progression. Higher expression of Cyclin D1 was strongly correlated with advanced tumor stage and positive lymph node metastasis in HNSCC [12]. A recent systemic review also demonstrated that CCND1 amplification or Cyclin D1 overexpression was significantly associated with HNSCC progression and malignant transformation of potentially malignant disorders [13]. Positive PAI-1 membrane expression was demonstrated to be an independent risk factor for local disease relapse of oral squamous cell carcinoma [14]. In addition, high PAI-1 level was strongly associated with aggressive clinicopathological parameters and unfavorable survival in HNSCC [15]. XRCC1 polymorphisms are closely associated with the risk and survival of HNSCC [16]. In addition, reduced XRCC1 levels has been found in HNC patients compared to normal controls [17], suggesting XRCC1 might play a tumor suppressive role in HNSCC. Mammalian/mechanistic target of rapamycin (mTOR) plays a critical role in tumorigenesis of HNSCC and serves as a molecular target for HNSCC [18, 19]. Raptor acts as scaffold protein and is important for maintaining the activation of mTOR [20]. HER3 is widely recognized as tumor promotor in HNSCC [21, 22]. Brand et al have demonstrated that HER3 was overexpressed in HPV positive HNSCC, and its upregulation was associated with worse overall survival in patients with pharyngeal cancer [21]. HER3 lacks the intrinsic tyrosine kinase activity and it is frequently phosphorylated by other receptor tyrosine kinases (RTKs). Phosphorylation of HER3 is important for activating oncogenic signaling, such as the PI-3K/Akt pathway and Src kinase [23]. Currently, there are at least 9 potential tyrosine phosphorylation sites in the carboxy-terminal tail of HER3 and HER3_pY1289 (HER3 phosphorylated at the tyrosine residue of 1289) is one of them. Therefore, HER3 upregulation in HNSCC does not definitely indicate that HER3_pY1289 is overexpressed in HNSCC, which was the major reason for choosing HER3_pY1289 for validation. To the best of our knowledge, this is the first study to demonstrate high HER3_pY1289 expression is associated with unfavorable prognosis of HNSCC.

The results of the stratified survival analysis of the validation cohort are not consistent with the discovery cohort in several subgroups of patients, including age>=60 years, female, G3-G4 and stage I-II. One possible reason accounting for the inconsistencies of the stratified survival analysis between the discovery and validation cohort is that the sample size in the subgroups are too small. For instance, for the validation cohort, there are only 44 patients, 36 patients and 29 patients in the female subgroup, G3-G4 subgroup and stage I-II subgroup, respectively. It is very difficult to reach a statistical significance due to the small sample size, and variations in a few samples can greatly affect the results. Therefore, increasing the sample size might reduce the inconsistency. For example, there are 84 patients in the age>=60 subgroup of the validation cohort. Although the *p* value (*p*=0.094) is larger than 0.05, we can still observe the trend that the HNSCC patients in the high-risk group suffered worse OS than those in the low risk group.

Four clinicopathologic parameters (age, gender, tumor grade and TNM stage) are chosen into the univariate and multivariate analyses. There are two reasons for choosing these features. Firstly, age, gender, tumor grade and TNM stage are important clinical variables for HNSCC. Secondly, most HNSCC patients in the TCGA dataset have data for these clinicopathological parameters. If other clinicopathological parameters are selected, most HNSCC cases will be removed. For instance, only 56 out of 346 cases in the TCPA cohort have the results of the HPV test. Therefore, it is unappropriated to include the HPV status into the univariate and multivariate models. In that case, the sample size (only around 20-30 patients in both the discovery and validation cohort) would be very small, which might significantly affect the results of univariate and multivariate analyses. In addition, the results of univariate and multivariate analyses might be not robust and reliable due to the small sample size.

We have also analyzed the associations between the protein-based prognostic signature and the common clinicopathological parameters of HNSCC (age, gender, tumor grade, and TNM stage), and no significant correlation is observed (data not shown). For other clinical features and molecular characteristics, the TCGA HNSCC dataset provides no or little information on these parameters. Further studies with more detailed information are warranted to explore the clinical significance of the protein-based prognostic signature.

There were several limitations for our current study. Firstly, the predictive power of the protein-based risk signature was only evaluated in TCPA validation cohort. Validation of the 5-protein prognostic model in other independent cohorts is very important for confirming its robustness. RPPA is used to determine the expression level of proteins in the HNSCC tissues for the TCPA cohort. RPPA is a high-throughput antibody-based technique with the procedures similar to that of western blots [24]. Thus, the IHC staining results are not suitable for validating the protein-based prognostic signature which is built on the RPPA methodology. Instead, RPPA should be performed to validate our 5-protein prognostic model in large-scale independent patient cohorts. Secondly, it is important to optimize the risk score formula to minimize the deviations in the tumor samples across different independent studies.

Collectively, we have developed a robust protein-based risk signature for accurately predicting the clinical outcome of HNSCC, which might contribute to the improvement of individualized treatments.

## MATERIALS AND METHODS

### Public data source

The proteome data of HNSCC cohort (level 4 data) which includes 346 HNSCC samples were downloaded from the TCPA portal (https://tcpaportal.org/tcpa/).

The clinical information of HNSCC cases were obtained from The National Cancer Institute Genomic Data Commons (NCI-GDC) (https://gdc.cancer.gov/). As the overall survival (OS) time was missing in one case, the remaining 345 HNSCC patients were subjected to the subsequent analysis.

### Construction of a protein-based prognostic signature based on the TCPA discovery cohort

The HNSCC dataset was randomly split into the discovery cohort (n=172) and validation cohort (n=173) using a computer-generated random sequence. The proteins that strongly associated with the OS of HNSCC patients were determined with the univariate cox proportional hazards regression analysis. The LASSO regression analysis was performed to identify the most optimal OS-associated proteins into the multivariate cox proportional hazards regression model. The multivariate analysis was used to determine the prognosis-related proteins and their coefficients. A risk score for each patient was calculated as the sum of each protein's score, which was obtained by multiplying the expression level of the protein and its corresponding coefficient. The TCPA discovery cohort was divided into high-risk group and low-risk group with the median value of the risk scores. The differences in OS and the OS stratified by clinicopathological parameters were compared between high and low-risk group.

### Validation of the protein based-prognostic signature using the TCPA validation cohort

Similarly, the TCPA validation cohort was divided into high-risk group and low-risk group with the same cut-off value in the TCPA discovery cohort. The OS and the OS stratified by clinicopathological parameters were compared between high and low-risk group.

### Nomogram model construction

The risk signature, age, gender, TNM stage and tumor grade were used to construct a nomogram model. Calibration curves were used to determine the agreement between model prediction outcome and actual outcome for one-year OS and there year OS.

### Tissue samples and IHC analysis

One hundred and twelve formalin-fixed paraffin-embedded (FFPE) tissue specimens and the corresponding clinical information were obtained from the Stomatological Hospital, Southern Medical University (SMUSH cohort). All the HNSCC cases were pathologically confirmed. The detailed information of the patient cohort was summarized in Table 1. This study was approved by the Institutional Research Ethics Committee at the Stomatological Hospital, Southern Medical University. Written informed consent was obtained from all patients for the using their tissue samples.

**Table 1:** The clinical information of the SMUSH cohort.

**Clinicopathological features**	**Number**
**Age**	
Mean ± SD	60.54 ± 8.13
**Gender, n (%)**	
Male	84 (75.00%)
Female	28 (25.00%)
**Pathological diagnosis**	
Squamous cell carcinoma	112 (100%)
**Tumor grade**	
G1	48 (42.86%)
G2	42 (37.50%)
G3	22 (19.64%)
G4	0 (0.00%)
**TNM stage**	
Stage I	19 (16.96%)
Stage II	43 (38.39%)
Stage III	28 (25.00%)
Stage IV	22 (19.64%)
**Lymph node metastasis**	
Negative	47 (41.96%)
Positive	65 (58.04%)
**Distant metastasis**	
No	110 (98.21%)
Yes	2 (1.79%)
**HPV infection**	
Negative	66 (70.21%)
Positive	28 (29.79%)

For IHC analysis, FFPE tissue sections were deparaffinized by sequential washing with xylene, 100% ethanol, 95% ethanol, 80% ethanol and PBS. Followed by quenching with 0.3% H_2_O_2_ in methanol for 5 min, the slides were blocked with 5% BSA for 30 min. Then the sections were incubated with HER3_pY1289 primary antibody (1:200, Cell Signaling Technology, Danvers, MA, USA) overnight at 4 °C. After washing with PBS, the slides were incubated with horseradish peroxidase (HRP)-conjugated secondary antibody for 2 h at room temperature. For the quantitative analysis, the IHC score of HER3_pY1289 was obtained by multiplying the staining intensity (on a scale of 0-3: negative = 0, weak = 1, moderate = 2, and strong = 3) and the percentage of cells stained (on a scale of 0-4: 0 = 0%, 1 = 1%-25%, 2 = 26%-50%, 3 = 51%-75%, and 4 = 76%-100%).

### Cell culture and siRNA transfection

The HNSCC cell lines SCC1 and SCC23 were cultured in Dulbecco's modified eagle medium (DMEM) supplemented with 10% fetal bovine serum, penicillin (100 U/mL), and streptomycin (100 μg/mL). Cultures were maintained at 37 °C in an atmosphere containing 95% air and 5% CO_2_. Based on the manufacturer’s manual, the cells were transfected with siRNAs targeting HER3 (siHER3 #1, siHER3 #2) and control siRNA (siCTRL) using the Lipofectamine® RNAiMAX Transfection Reagent (Invitrogen, Carlsbad, CA, USA).

### Western blotting

Equal amount of protein samples was loaded and separated on a 4-12% Bis-Tris NuPAGE gel (Invitrogen) and transferred onto a nitrocellulose membrane using a Trans-blot SD semi-dry transfer cell (Bio-Rad Laboratories Inc., Hercules, CA, USA). The membranes were blocked in TBST buffer containing 5% nonfat milk for 1 h at room temperature. Then the membranes were incubated with HER3_pY1289 primary antibody (1:500, CST) and HER3 antibody (1:500, CST) overnight in the cold room. After rinsing in TBST for three times, the membranes were incubated with HRP-link secondary antibody for 1 h at room temperature. ECL kit (Beyotime Biotech, Shanghai, China) was applied to visualize the bands.

### MTT assay

The siHER3 treated cells and the control cells were seeded into 96-well plates at a density of 3000 cells/well. At the indicated time points (24h, 48h, 72h and 96h), 20 μL MTT solution (Sigma-Aldrich, St. Louis, MO, USA) was added to each well and the plates were incubated for 4 h at 37 °C. After removing the supernatant, the formazan products were dissolved by adding 200 μL dimethyl sulfoxide. Followed by shaking for 30s, the absorbance was determined using a microplate reader (Tecan, Mannedorf, Switzerland).

### EdU (5-ethynyl-2′-deoxyuridine) assay

EdU assay was conducted with the Click-iT™ EdU Cell Proliferation Kit for Imaging (Invitrogen) based on the manufacturer’s protocol. Briefly, EdU was added to the cells at the final concentration of 10 μM and incubated for 2 h at 37°C. After removing the supernatant, the cells were fixed with 3.7% formaldehyde in PBS for 20 min at room temperature. The fixative was removed and 0.5% Triton® X-100 (Sigma-Aldrich) in PBS was added to increase the permeability of the cellular membrane. The cells were stained with 1 × Click-iT reaction cocktail for 30 min at room temperature and protected from light. Hoechst 33342 dye was then used for nuclear staining. Images were obtained under a confocal laser scanning microscope (Olympus, Center Valley, PA, USA) and at least four random fields per well were photographed for data analysis.

### Transwell matrigel invasion assay

After starvation for overnight, the cells were washed two times with PBS and then resuspended in pure DMEM. Then 5 × 10^5^ cells were added to the upper chamber of transwell matrigel invasion inserts (BD Biosciences. Bedford, MA, USA). The lower chamber is filled with 1 mL of complete growth medium. After 24 h, a cotton swab was used to remove the cells remaining on the upper surface of the membrane. Then the cells that had invaded through the membrane were fixed by 3.7% formaldehyde and stained with the crystal violet. At least four random fields per insert were captured for data analysis.

### Statistical analysis

The statistical analysis was performed with GraphPad Prism 8.0 (GraphPad Software Inc., San Diego, CA, USA). The data of *in vitro* experiments were expressed as the mean ± standard deviation, and analyzed by the one-way ANOVA. For the SMUSH cohort, the median value of the IHC scores was used to divide the HNSCC patients into the high IHC score group and low IHC score group. The OS difference between high and low IHC score group was determined by the Kaplan-Meier method and log-rank test. All statistical analyses were two-sided. A *p* value of less than .05 was considered statistically significant.

## Supplementary Material

Supplementary Tables
