# Supplementary material for: Development of a five-protein signature for predicting the prognosis of head and neck squamous cell carcinoma
Source: Aging (Albany NY). 2020 Oct 13;12(19):19740–55. doi: 10.18632/aging.104036 (PMC7732293; doi:10.18632/aging.104036)
Supplement: Supplementary Tables [file aging-12-104036-s001..pdf]

SUPPLEMENTARY TABLES

Supplementary Table 1. The overall survival associated proteins identified by univariate analysis.

Protein ID	HR	HR.95L	HR.95H	p value
CYCLIND1	6.75	2.69	16.93	0.00005
CMET_pY1235	10.33	2.83	37.70	0.00041
IRS1	3.20	1.53	6.71	0.00200
HER3_pY1289	4.69	1.73	12.74	0.00244
XRCC1	0.29	0.13	0.67	0.00379
MSH6	0.47	0.27	0.81	0.00633
BID	2.59	1.30	5.15	0.00673
BRAF_pS445	0.29	0.12	0.72	0.00748
RAPTOR	4.02	1.44	11.21	0.00791
ECADHERIN	0.79	0.66	0.94	0.00805
PAI1	1.41	1.09	1.82	0.00911
BAP1C4	0.54	0.33	0.87	0.01198
BETACATENIN	0.75	0.60	0.94	0.01329
ANNEXINVII	0.26	0.09	0.76	0.01413
PAXILLIN	1.68	1.11	2.55	0.01500
PDCD4	0.68	0.50	0.93	0.01680
CAVEOLIN1	1.29	1.04	1.60	0.01995
BIM	0.54	0.32	0.92	0.02387
SYK	0.61	0.40	0.94	0.02453
CYCLINE2	0.43	0.20	0.90	0.02490
TUBERIN_pT1462	0.26	0.08	0.87	0.02840
PTEN	0.61	0.39	0.95	0.02854
PCNA	0.38	0.16	0.91	0.02941
P70S6K1	0.41	0.18	0.92	0.03045
EEF2K	0.71	0.52	0.97	0.03199
CLAUDIN7	0.72	0.53	0.98	0.03439
FIBRONECTIN	1.60	1.03	2.50	0.03684
PEA15	2.18	1.05	4.54	0.03761
PI3KP110ALPHA	0.48	0.24	0.97	0.03980
ADAR1	0.39	0.16	0.97	0.04365
NF2	2.03	1.02	4.05	0.04473
MSH2	0.46	0.21	0.99	0.04851
RAD51	4.11	1.00	16.81	0.04933

Supplementary Table 2. The clinical information of the discovery cohort.

Clinicopathological features	Number
**Age**	
>=60	78 (52.35%)
<60	71 (47.65%)
**Gender, n (%)**	
Male	103 (69.13%)
Female	46 (30.87%)
**Pathological diagnosis**	
Squamous cell carcinoma	149 (100%)
**Tumor grade**	
G1	17 (11.41%)
G2	95 (63.76%)
G3	37 (24.83%)
G4	0 (0.00%)
**TNM stage**	
Stage I	10 (6.71%)
Stage II	28 (18.79%)
Stage III	29 (19.46%)
Stage IV	82 (55.03%)

Supplementary Table 3. The clinical information of the validation cohort.

Clinicopathological features	Number
**Age**	
>=60	84 (54.90%)
<60	69 (45.10%)
**Gender, n (%)**	
Male	109 (71.24%)
Female	44 (28.76%)
**Pathological diagnosis**	
Squamous cell carcinoma	153 (100%)
**Tumor grade**	
G1	25 (16.34%)
G2	92 (60.13%)
G3	36 (23.53%)
G4	0 (0.00%)
**TNM stage**	
Stage I	4 (2.61%)
Stage II	25 (16.34%)
Stage III	28 (18.30%)
Stage IV	96 (62.75%)
